# Serum reactivity to citrullinated protein/peptide antigens and left ventricular structure and function in the Multi-Ethnic Study of Atherosclerosis (MESA)

**DOI:** 10.1371/journal.pone.0291967

**Published:** 2023-10-24

**Authors:** Jan M. Hughes-Austin, Ronit Katz, Darcy S. Majka, Michael H. Criqui, William H. Robinson, Gary S. Firestein, W. Gregory Hundley, Joachim H. Ix

**Affiliations:** 1 Department of Orthopaedic Surgery, University of California, San Diego, La Jolla, California, United States of America; 2 Department of Obstetrics & Gynecology, University of Washington, Seattle, Washington, United States of America; 3 Division of Rheumatology, DuPage Medical Group, Chicago, Illinois, United States of America; 4 Department of Family Medicine and Public Health, University of California, San Diego, La Jolla, California, United States of America; 5 Division of Immunology and Rheumatology, Stanford University, Stanford, California, United States of America; 6 VA Palo Alto Health Care System, Palo Alto, California, United States of America; 7 Division of Rheumatology, Allergy, and Immunology, Department of Medicine, University of California, San Diego, La Jolla, California, United States of America; 8 Virginia Commonwealth University, Richmond, Virginia, United States of America; 9 Division of Nephrology-Hypertension, Department of Medicine, University of California, San Diego, La Jolla, California, United States of America; Kyoto University Graduate school, JAPAN

## Abstract

**Background:**

Antibodies to citrullinated protein antigens have been linked to altered left ventricular (LV) structure and function in patients with rheumatoid arthritis (RA). Serum reactivity to several citrullinated protein/peptide antigens has been identified in RA, which are detectable years before RA onset and in individuals who may never develop RA. Among community-living individuals without heart failure (HF) at baseline in the Multi-Ethnic Study of Atherosclerosis (MESA), we investigated associations between serum reactivity to citrullinated protein/peptide antigens, LV mass, LV ejection fraction (LVEF), and incident HF.

**Methods:**

Among 1232 MESA participants, we measured serum reactivity to 28 different citrullinated proteins/peptides using a multiplex bead-based array. Each antibody was defined as having extremely high reactivity (EHR) if >95^th^ percentile cut-off in MESA. Number of EHR antibody responses to citrullinated protein/peptide antigens were summed for each participant (range 0–28). LV mass(g) and LVEF(%) were measured on cardiac MRI. Associations between EHR antibodies and LV mass and LVEF were evaluated using linear regression. Cox proportional hazards models were used to evaluate associations between EHR antibodies and incident HF during 11 years of follow-up, adjusting for age, gender, race/ethnicity, smoking status, systolic blood pressure, use of anti-hypertensive medications, self-reported arthritis, IL-6, body surface area, and estimated glomerular filtration rate.

**Results:**

Mean age was 65±10, 50% were female, 40% were White, 21% were Black, 26% were Hispanic/Latino, and 14% were Chinese. Twenty-seven percent of MESA participants had extremely high reactivity to ≥ 1 citrullinated protein/peptide antigen. In fully adjusted analysis, every additional EHR antibody was significantly associated with 0.1% lower LVEF (95% CI: -0.17%, -0.02%). No association was observed with LV mass (β per additional EHR antibody) = 0.13±0.15 (p = 0.37)). Neither the presence nor number of EHR antibodies was associated with incident HF during follow-up (HR per additional EHR antibody = 1.008 (95% CI: 0.97, 1.05)).

**Conclusion:**

Greater number of extremely highly reactive antibodies was associated with lower LVEF, but not with LV mass or incident HF. Thus, serum reactivity to citrullinated protein/peptide antigens was associated with subtle subclinical changes in myocardial contractility, but the significance in relation to clinically apparent HF is uncertain.

## Introduction

Among individuals with rheumatoid arthritis (RA), risk for heart failure is 70% higher compared to the general population [1]. Given that traditional cardiovascular disease (CVD) risk factors do not explain the higher CVD risk or the higher rates of heart failure in patients with RA [1–6], it is thought that RA-related autoantibodies may predispose individuals to early subclinical cardiovascular changes ultimately resulting in these adverse cardiac outcomes [7, 8]. It has been proposed that RA begins with a genetic predisposition to develop autoantibodies in response to an environmental trigger [9]. Once triggered, the development of autoantibodies is associated with higher levels of inflammatory cytokines which may also drive CVD [10–12]. Less is known about the direct associations between RA-related autoantibodies and pre-clinical cardiac structure and functional changes.

Geraldino-Pardilla and colleagues investigated associations between individual antibodies to citrullinated protein antigens (ACPA) and left ventricular structure and function in RA patients [13]. They found that antibodies to citrullinated vimentin and fibrinogen were associated with higher left ventricular mass index (LVMI) independent of gender, smoking status, hypertension, and low density lipoprotein-cholesterol [13]. While the LVMI values among RA patients that had high ACPA levels still fell within the normal anatomic range, the authors proposed that these higher LVMI may indicate progression toward heart failure development.

ACPA are known to become detectable several years before clinical manifestation of RA, and are also detectable in some seemingly healthy individuals who do not develop RA. ACPA reactivity to several different citrullinated and non-citrullinated proteins/peptides has been identified in RA as well as during the ‘preclinical’ period of RA [14–21]. Serum reactivity to citrullinated proteins/peptides in a community-living population is unclear, although prior studies have reported a 1% ACPA prevalence in study populations of RA-free individuals in Turkey and Japan [22, 23]. Moreover, the clinical consequences of serum reactivity to citrullinated antigens in relation to subclinical and clinical heart failure is uncertain. Thus, we measured serum antibodies to 28 distinct citrullinated proteins/peptides in a large multi-ethnic community-living population, the Multi-Ethic Study of Atherosclerosis. We hypothesized that serum reactivity to these citrullinated proteins/peptides would be detectable in some community-living individuals, and such individuals would have lower LV ejection fraction (EF), greater LV mass, and higher risk of clinically apparent heart failure, independent of traditional risk factors.

## Materials and methods

### The multi-ethnic study of atherosclerosis

The Multi-Ethnic Study of Atherosclerosis (MESA) is a multi-center prospective cohort study that enrolled 6814 participants from six field centers across the US [Baltimore, MD; Chicago, IL; Los Angeles, CA; New York, NY; St. Paul, MN; and Winston-Salem, NC] to evaluate race/ethnic differences in risk factors for subclinical and clinical CVD. This study started in July 2000–2002 and included individuals who were Black, Chinese, Hispanic/Latino, and White Americans and were aged ≥ 45 years with no clinically apparent CVD. Individuals were excluded if they had clinical CVD, including physician-diagnosed myocardial infarction, angina, stroke, transient ischemic attack, or heart failure; use of nitroglycerine; or current atrial fibrillation, or had undergone a procedure related to CVD. During clinic visits, participants underwent an extensive physical exam, with computed tomography (CT) and magnetic resonance (MR) imaging, had fasting blood drawn and stored, and completed questionnaires. More extensive details regarding study design, methods, examinations, and data collection have been previously published [24]. All research was performed in accordance with relevant guidelines and regulations. All participants provided informed written consent, and all MESA sites obtained Institutional Review Board approval [Wake Forest University, Columbia University, Johns Hopkins University, University of Minnesota, Northwestern University, University of Los Angeles, and University of Washington Institutional Review Boards]. This ancillary study was also approved by the UC San Diego Human Research Protection Program.

### Study population

Our study population consisted of participants in the MESA Abdominal Aortic Calcium Ancillary Study, which collected abdominal CT scans on 1968 individuals randomly selected from 5 of 6 MESA field centers [all except Baltimore, MD] between August 2002 and September 2005. Characteristics between this ancillary study cohort and the overall MESA cohort did not differ. Among these 1968 individuals, 1647 had complete measurements for all antibodies measured.

We used MRI data from the baseline exam. In merging the MRI data with the antibody and clinical data, 415 were missing MR images. Thus, combining individuals with complete antibody data, complete MRI data, and complete covariate data totaled to 1232 for our analysis dataset.

### Testing of antibodies to citrullinated and non-citrullinated proteins/peptides

A multiplex platform [25] used autoantibodies to 28 citrullinated autoantigens and 10 native proteins/peptides [26] (S1 Table). These citrullinated proteins/peptides were selected based on antigens identified in immune complexes isolated from synovial tissue of patients with RA as well as antigens reported in the literature [27]. The array uses a custom Bio-Plex^™^ (BioRad, Hercules, CA) bead-based autoantibody assay in which antigens are conjugated to spectrally-distinct beads. Protein antigens are coupled to beads using N-hydroxysuccinimide ester chemistry, and peptide antigens synthesized with C- terminal biotin (by Fmoc chemistry) and coupled to avidin-coated beads. Pooled beads are mixed with serum samples and diluents and incubated at room temperature. After washing, anti-human IgG antibody conjugated to phycoerythrin (PE) was added to the dyed beads and incubated at room temperature. After another wash, the bead mixture was passed through a laser detector (Luminex 200, Austin, TX) that identified beads based on the fluorescence of the dyes. The amount of antibody bound to each bead was determined by the fluorescence of PE. This assay has yielded highly reproducible results with 7-fold intra-assay coefficient of variance (CV) of 0.9–6.8% and 14-fold inter-assay CV ranging from 5.9–19.5% (over 90% of beads yielding inter-assay variances of < 12%) [26].

Measurements of rheumatoid factor (RF) IgM and IgG as well as anti-cyclic citrullinated peptide (anti-CCP2) antibodies had previously been measured in MESA, and were evaluated in parallel with antibodies to citrullinated and non-citrullinated proteins/peptides in this current investigation [28]. Inter- and intra-assay CVs for these antibodies were <10% [28].

### Left ventricular structure and function

Our main outcome variables were left ventricular (LV) mass, measured in grams, and left ventricular (LV) ejection fraction, measured as a percent. These variables were based on measurements from MRI which followed a protocol based on the fast gradient echo MRI pulse sequence to obtain images of the heart [29]. Briefly, cardiac MR examinations were comprised of short- and long-axis cine images, phase contrast images of the aorta, and black blood aorta images [29]. All images were acquired during short breath-holding (12–15 sec) at resting lung volume [29]. LV mass was determined by the sum of the myocardial area times the slice thickness plus image gap in the end-diastolic phase multiplied by the specific gravity of the myocardium (1.05 g/mL). LV ejection fraction was calculated as LV stroke volume divided by LV end-diastolic volume multiplied by 100, where LV stroke volume was calculated as the difference between LV end-diastolic volume and LV end-systolic volume [29]. For secondary analysis, we evaluated LV end-diastolic and LV end-systolic volumes individually.

### Incident heart failure outcomes

We also investigated associations between serum reactivity to citrullinated protein/peptide antigens and incident heart failure. These events were adjudicated by a committee in MESA, and included both definite and probable heart failure, defined by heart failure symptoms, and by physician diagnosis with medical treatment. Specifically, definite and probable heart failure required clinical symptoms, e.g., shortness of breath, and signs e.g., edema since asymptomatic disease was not an endpoint. Probable heart failure required a physician diagnosis and medical treatment for heart failure. Definite heart failure required pulmonary edema/congestion on chest x-ray and/or dilated ventricle or poor LV function by echocardiography or ventriculography, or evidence of LV diastolic dysfunction [30, 31].

### Covariates

Standardized questionnaires were used to collect participant demographics, ethnicity, medical history, self-reported arthritis, and medication usage including use of anti-hypertensive medications. Cigarette smoking was classified as never, current, or former, and was dichotomized in analyses to ‘ever’ versus ‘never.’

Resting blood pressure was measured three times in the seated position using a Dinamap automated sphygmanometer, and the average of the second and third readings was used for this analysis. Height and weight were measured with participants wearing light clothing and no shoes. Body mass index (BMI) was calculated as weight (kg)/height (m^2^). Body surface area (BSA) was calculated using the Mostellar formula [32]. Estimated glomerular filtration rate (eGFR) was calculated using the creatinine-cystatin-C equation in mL/min/1.73m^2^ [33].

Blood samples were obtained after a 12-hour fast to measure biomarkers. IL-6 concentrations were measured by ultrasensitive ELISA (Quantikine HS Human IL-6 Immunoassay; R&D Systems, Minneapolis, MN, USA). Average analytic coefficient of variation for this assay was 9.3%.

### Statistical analysis

We classified each antibody using 95^th^ percentile cutoffs, where individuals in the top 5% of each individual antibody were considered having extremely high reactivity to that antigen. Then we categorized individuals as having extremely high reactivity (EHR) or within normal reactivity (WNR) for antibodies to citrullinated proteins/peptides; and subsequently counted the number of EHR antibodies for each individual (range 0–28).

For descriptive statistics comparing continuous variables between individuals with extremely high reactivity and within normal reactivity to citrullinated proteins/peptides, we used Student’s t-tests or Wilcoxon rank-sum tests for normally and non-normally distributed variables, respectively; and chi-square tests to compare categorical variables. For the cross-sectional analysis of EHR antibodies with LV mass and ejection fraction and LV end systolic and diastolic volume, we used analysis of covariance (ANCOVA). For the association between number of EHR antibodies and LV mass and ejection fraction, and LV end systolic and diastolic volume, we used multivariable linear regression. For Anti-CCP2, RF-IgA, and RF-IgM antibodies, we log-transformed these autoantibody concentrations and analyzed them per standard deviation (SD) change in multivariable linear regression.

For our longitudinal analysis investigating associations of EHR, anti-CCP2, RF-IgA, and RF-IgM antibodies with incident heart failure, we considered their follow-up time from August 2002 until an event or until December 2014, and used Cox proportional hazards regression.

All analyses were adjusted for age, gender, race/ethnicity, smoking status, systolic blood pressure, use of anti-hypertensive medications, self-reported arthritis, IL-6, body surface area (BSA) and estimated glomerular filtration rate (eGFR). We tested for the effect of gender and race/ethnicity on associations between EHR antibodies and LV structure and function as well as incident HF. SAS version 9.4 (SAS Systems, Inc., Cary, NC) was used for statistical analysis, and p-values < 0.05 were considered statistically significant.

## Results

Among the 1647 MESA participants included in this study, the average age was 65 ± 10, 40% were White, and 50% were women. There were 444 (27%) who had extremely high reactivity to at least 1 citrullinated protein/peptide antigen, and 1203 (73%) who had within normal reactivity to all 28 citrullinated proteins/peptides. We found no significant differences between MESA participants with extremely high reactivity versus within normal reactivity except that those with at least 1 EHR response to a citrullinated antigen were more likely to have detectable anti-CCP2 antibodies and had higher IL-6 concentrations (Table 1).

**Table 1:** Population characteristics of 1647 MESA participants stratified by having extremely high reactivity to at least one citrullinated protein/peptide versus having within normal reactivity to all citrullinated proteins/peptides.

	Total	Extremely High Reactivity (EHR)	Within Normal Reactivity (WNR)	p-value
	(n = 1647)	(n = 444)	(n = 1203)	
Age, years	65.3 (9.7)	65.8 (10.1)	65.1 (9.5)	0.15
Gender, % female	50	51	50	0.68
Race/Ethnicity				0.12
% White, 1	40	39	40	
% Chinese, 2	14	11	15	
% Black, 3	21	23	20	
% Hispanic/Latino, 4	26	28	25	
BMI, kg/m^2^	28.1 (5.2)	28.0 (5.1)	28.4 (5.5)	0.26
Systolic Blood Pressure, mmHg	124.0 (21.1)	124.3 (21.0)	123.9 (21.1)	0.71
Diastolic Blood Pressure, mmHg	70.0 (9.9)	70.2 (9.8)	69.9 (10.0)	0.61
Hypertension, %	46	47	46	0.67
Fasting blood glucose, mg/dL	99.0 (27.8)	99.5 (29.4)	98.8 (27.3)	0.70
Diabetes, %	15	15	14	0.69
RF Positive, %	20	22	19	0.16
CCP2 Positive, %	**1.6**	**3.8**	**0.8**	**<0.0001**
LV Mass, g	146.1 (38.8)	147.2 (37.6)	145.7 (39.3)	0.53
LV Ejection Fraction, %	69.3 (7.4)	69.0 (7.1)	69.5 (7.5)	0.17
eGFR, mL/min/1.73m^2^	78.5 (17.9)	77.2 (17.0)	78.9 (18.1)	0.09
ACR, mg/g median (p25, p75)	6.2 (3.7, 12.8)	6.3 (3.7, 11.8)	6.2 (3.7, 13)	0.54*
CKD, %	9	10	9	0.47
Fibrinogen, mg/dL	434.2 (93.0)	440.6 (91.6)	431.9 (93.5)	0.09
TNFa, pg/mL, median (p25, p75)	4.6 (3.4, 6.4)	4.8 (3.5, 6.4)	4.6 (3.4, 6.3)	0.24*
IL-6, pg/mL, median (p25, p75)	1.8 (1.2, 2.9)	**1.9 (1.3, 3.1)**	**1.8 (1.2, 2.8)**	**0.004** *
CRP, mg/L, median (p25, p75)	1.4 (0.7, 3.3)	1.5 (0.8, 3.6)	1.4 (0.7, 3.3)	0.07*
Triglycerides, mg/dL median (p25, p75)	112.0 (76, 162)	111 (75, 161)	112 (77, 163)	0.34*
HDL cholesterol, mg/dL	51.3 (15.5)	51.2 (15.4)	51.4 (15.5)	0.87
LDL cholesterol, mg/dL	110.7 (31.5)	110.1 (32.6)	110.9 (31.1)	0.65
Current smoker, %	11	10	11	0.54
Alcohol use, %	50	49	51	0.47
Lipid lowering medications, %	22	21	22	0.73
Anti-hypertensive medications, %	42	43	41	0.57
Education greater than high school, %	49	46	50	0.18
Self-reported arthritis, %	11	11	11	0.92

BMI = body mass index, RF = rheumatoid factor, CCP2 = cyclic citrullinated peptide, eGFR = estimated glomerular filtration rate, ACR = albumin creatine ration, CKD = chronic kidney disease, TNF-a = tumor necrosis factor alpha, IL-6 = interleukin 6, CRP = c-reactive protein, HDL = high density lipoprotein, LDL = low density lipoprotein

* Wilcoxon rank-sum tests were used to compare medians

Among the 1232 MESA participants with antibody, MRI, and clinical data, 362 (29%) had extremely high reactivity to at least 1 citrullinated protein/peptide antigen. Compared to those who had within normal reactivity to citrullinated protein/peptide antigens, those with extremely high reactivity to at least 1 citrullinated antigen had similar LV mass and LVEF (Table 1). Among these individuals, the median (25^th^, 75^th^ percentile) number of EHR antibodies was 2 (1, 6). When evaluating the 362 individuals with at least 1 EHR antibody response to a citrullinated protein/peptide antigen, the number of EHR antibodies were associated with lower LV EF. In fully adjusted analysis of this cohort, each additional EHR antibody response to a citrullinated antigen was associated with 0.1% lower left ventricular ejection fraction (95% CI: -0.17%, -0.02%, p = 0.01). Among the 362 with at least 1 EHR antibody response to a citrullinated protein/peptide antigen, greater number of EHR antibodies was not significantly associated with LV mass (p = 0.37). There were also no significant associations between the number of EHR antibodies with left ventricular end diastolic and end systolic volumes (Table 2). In supplemental analysis, we evaluated associations between individual autoantigens, citrullinated and non-citrullinated, and found that none were associated with LV mass; and that antibodies to citrullinated Histone 2B as well as 3 of the native autoantigens (Filaggrin _48–65 arg2 v1 cyclic_, Histone 2A, and Histone 2B) were significantly associated with LV ejection fraction (S2 Table).

**Table 2:** Associations between extremely highly reactive antibodies, left ventricular (LV) ejection fraction, LV mass, LV end diastolic volume, and LV end systolic volume in MESA participants with extremely high reactivity to ≥ 1 citrullinated protein/peptide antigen and free of RA.

	Extremely High Reactivity (EHR) vs Within Normal Reactivity (WNR) Antibody Responses		Number of Extremely High Reactivity (EHR) Antibody Responses	
	β (SE)	p-value	β (SE)	p-value
**LVEF Unadjusted**	-0.42 (0.46)	0.36	-0.07 (0.04)	0.09
**LVEF Adjusted** *	-0.35 (0.42)	0.41	**-0.10 (0.04)**	**0.01**
**LV Mass Unadjusted**	1.63 (2.41)	0.50	0.02 (0.18)	0.90
**LV Mass Adjusted** *	0.86 (1.60)	0.59	0.13 (0.15)	0.37
**LV End Diastolic Volume Unadjusted**	-0.64 (1.90)	0.74	-0.15 (0.17)	0.37
**LV End Diastolic Volume Adjusted** *	-0.89 (1.48)	0.55	-0.003 (0.14)	0.98
**LV End Systolic Volume Unadjusted**	0.31 (1.04)	0.76	0.06 (0.10)	0.51
**LV End Systolic Volume Adjusted** *	0.14 (0.85)	0.87	0.14 (0.08)	0.07

*Adjusted for age, gender, race/ethnicity, smoking status, systolic blood pressure, use of anti-hypertensive medications, self-reported arthritis, IL-6, body surface area, and estimated glomerular filtration rate

In parallel assessment of anti-CCP2, RF-IgA, and RF-IgM antibodies with LV mass and LVEF, we found that neither anti-CCP2, RF-IgA, nor RF-IgM antibodies were associated with LV mass or LV ejection fraction in fully adjusted analysis (Table 3). With regard to LV end diastolic and systolic volume, we found that for every standard deviation higher anti-CCP2 level, LV diastolic volume was 2 units lower (β = -2.08 (0.86), p = 0.02) and LV end systolic volume was 1 unit lower (β = -0.94 (0.49), p = 0.06) in fully adjusted analysis. There were no associations between RF-IgA or RF-IgM antibodies with LV end diastolic or systolic volume.

**Table 3:** Associations of Anti-CCP2, RF-IgA, and RF-IgM antibodies with left ventricular (LV) ejection fraction, LV mass, LV end diastolic volume, and LV end systolic volume in MESA participants with extremely high reactivity to ≥ 1 citrullinated protein/peptide antigen and free of RA.

	Anti-CCP2 (per SD)		RF-IgA (per SD)		RF-IgM (per SD)	
	β (SE)	p-value	β (SE)	p-value	β (SE)	p-value
**LVEF Unadjusted**	0.22 (0.24)	0.36	-0.08 (0.22)	0.70	-0.03 (0.21)	0.88
**LVEF Adjusted**	0.16 (0.25)	0.50	-0.06 (0.21)	0.78	-0.06 (0.20)	0.76
**LV Mass Unadjusted**	-1.39 (1.28)	0.28	1.18 (1.16)	0.31	-0.003 (1.12)	1.00
**LV Mass Adjusted**	-0.76 (0.94)	0.42	-0.02 (0.81)	0.98	0.17 (0.76)	0.82
**LV End Diastolic Volume Unadjusted**	**-2.35 (1.01)**	**0.02**	-0.36 (0.91)	0.69	-0.38 (0.88)	0.67
**LV End Diastolic Volume Adjusted**	**-2.08 (0.86)**	**0.02**	-0.06 (0.75)	0.94	-0.33 (0.70)	0.64
**LV End Systolic Volume Unadjusted**	**-1.09 (0.56)**	**0.05**	-0.09 (0.50)	0.86	-0.11 (0.48)	0.82
**LV End Systolic Volume Adjusted**	**-0.94 (0.49)**	**0.06**	-0.03 (0.43)	0.94	-0.04 (0.40)	0.93

*Adjusted for age, gender, race/ethnicity, smoking status, systolic blood pressure, use of anti-hypertensives, self-reported arthritis, IL-6, body surface area, and estimated glomerular filtration rate

We next evaluated the associations of extremely highly reactive antibody responses with risk of incident heart failure. Among the 1641 participants with antibody measurements at baseline, 69 participants experienced incident HF during 10.7 ± 2.4 years mean follow-up. We found no significant associations between EHR vs WNR antibody responses with incident HF. Among the subset with 1 or more EHR antibody response to a citrullinated protein/peptide antigen, we also found no association between the number of EHR antibodies and incident heart failure (Table 4). In addition, 68 participants positive for RF-IgA, RF-IgM or anti-CCP2 antibodies experienced heart failure although there was not significant association of RF-IgA, RF-IgM, or anti-CCP2 antibodies with incident heart failure in our longitudinal analysis. There were no significant interactions with race/ethnicity or gender in all the analyses.

**Table 4:** Associations of extremely highly reactive (EHR) antibodies to citrullinated proteins/peptides, anti-CCP2, RF-IgA, and RF-IgM antibodies with incident heart failure in 1641 MESA participants over an average of 11 years.

	EHR vs WNR	Number of EHR antibodies	Anti-CCP2 (per SD)	RF IgA (per SD)	RF IgM (per SD)
	HR (95% CI)	HR (95% CI)	HR (95% CI)	HR (95% CI)	HR (95% CI)
**Event / Total N**	69 / 1641	69 / 1641	68 / 1627	68 / 1630	68 / 1630
**Event Proportion**	4.23	4.23	4.17	4.17	4.17
**Unadjusted**	1.10 (0.66–1.86)	1.02 (0.99–1.07)	1.07 (0.88–1.30)	1.23 (1.004–1.50)	1.02 (0.80–1.29)
**Age, gender, race/ethnicity adjusted**	0.95 (0.56–1.60)	1.01 (0.97–1.05)	1.05 (0.86–1.29)	1.11 (0.89–1.39)	0.98 (0.77–1.24)
**Fully adjusted** *	0.92 (0.54–1.58)	1.008 (0.97–1.05)	0.85 (0.55–1.32)	1.02 (0.79–1.31)	0.90 (0.70–1.15)

*Adjusted for age, gender, race/ethnicity, smoking status, systolic blood pressure, use of anti-hypertensives, self-reported arthritis, IL-6, body surface area, and estimated glomerular filtration rate

## Discussion

In this multi-ethnic community-dwelling population of individuals without CVD, extremely high reactivity to RA-related citrullinated protein/peptide antigens was detected in approximately one-third (27%) of the cohort. These individuals appeared markedly similar in respect to demographics and clinical characteristics compared to those who had within normal reactivity to citrullinated protein/peptide antigens, but had higher concentrations of anti-CCP2 antibodies and the inflammatory biomarker, IL-6. Among those who had extremely high reactivity to ≥1 citrullinated protein/peptide antigen, the number of these EHR antibodies was associated with lower left ventricular ejection fraction, but not with left ventricular mass, nor with incident heart failure over 11 years of follow-up. Neither extremely high reactivity to citrullinated antigens nor the number of EHR antibodies were significantly associated with left ventricular end systolic or diastolic volume. We suspect EHR antibodies to citrullinated protein/peptide antigens could contribute to subtle subclinical changes in the myocardium that are associated with lower EF, but given absent associations with incident heart failure, the clinical consequences of these associations are uncertain.

Extremely high reactivity to citrullinated protein/peptide antigens was present in 27% of MESA participants, which suggests that these individuals may have an underlying level of autoantibody-related inflammation. Prior studies in first-degree relatives of patients with RA have shown that having RA-related autoantibodies is associated with higher concentrations of inflammatory biomarkers including IL-6, as confirmed in our study [10]. First degree relatives of RA patients with RA-related autoantibodies were also more likely to have inflamed joints [34], even without having clinically apparent RA. Given these data, one-third of MESA participants have an autoantibody profile that could be related to higher concentrations of circulating inflammatory cytokines, and subsequently inflammatory-related health outcomes.

Among the subset of 362 of individuals who had extremely high reactivity to ≥1 citrullinated protein/peptide antigen, the number of EHR antibodies was significantly associated with lower left ventricular ejection fraction. The number of EHR antibodies, however, was not associated with greater LV mass, nor with incident heart failure. Supplemental analysis of individual antibodies to citrullinated and non-citrullinated antigens affirmed no associations with LV mass; and demonstrated an association of anti-citrullinated Histone 2B with LV ejection fraction. Three of the non-citrullinated antigens, Filaggrin _48–65 arg2 v1 cyclic_, Histone 2A, and Histone 2B were also associated with lower LV ejection fraction, suggesting that antibodies to histones, citrullinated and non-citrullinated, may play a role in left ventricular function and warrant further investigation. Given that prior work has shown serum reactivity to citrullinated and non-citrullinated proteins/peptides in the preclinical phase of RA [14–21], it is possible that these antibodies stimulate inflammation which may have long term effects to alter cardiac function. This could conceivably lead to higher risk of HF, albeit we did not detect such a relationship despite following participants for approximately 11 years after antibody measurement.

To better understand our finding of an association of greater number of EHR antibodies with lower left ventricular ejection fraction, we investigated associations of EHR antibodies with left ventricular end systolic and diastolic volumes. We found that neither having extremely high reactivity nor the number of EHR antibodies were significantly associated with these parameters, but the marginally significant association between number of EHR antibodies and left ventricular end systolic volume suggests that there are subtle ventricular changes that could lead to a lower ejection fraction. Further, our evaluation of anti-CCP2, RF-IgM, and RF-IgA antibodies revealed that neither RF-IgM nor RF-IgA were associated with any of the cardiovascular structural outcomes, nor incident HF. Anti-CCP2 was, however, inversely associated with LV end diastolic and systolic volumes.

To our knowledge, this is the first large-scale evaluation of the relationship of serum reactivity to citrullinated protein/peptide antigens with cardiovascular structure and function in a community-living population, not selected for having autoimmune disease or among relatives of those affected by autoimmunity. The inclusion of men and women, diverse race/ethnicity, high quality assessment of cardiac structure by MRI, and adjudication of clinical HF events during long-term follow-up are additional strengths. The study also has important limitations. We utilized MRI data from the baseline exam which was 2 years prior to Exam 2/3 where antibodies were measured, so we cannot conclude that extremely high reactivity to citrullinated protein/peptide antigens leads to myocardial changes, and instead only report that there were associations between antibodies to citrullinated proteins/peptides and myocardial function. Further, we did not have a formal diagnosis of RA for study participants. Due to the unknown RA status of MESA participants, we were not able to establish cut-offs for antibody positivity using receiver operating characteristic (ROC) curves, and instead followed the 95% cutoff for extremely high reactivity as defined in the 1987 American College of Rheumatology Criteria [35]. Thus, it is likely that some MESA participants were misclassified, which may have attenuated the associations between EHR antibodies, LV mass, and LV ejection fraction. We studied persons in the general population, so we believe that the prevalence of RA or other autoimmune diseases linked to cardiovascular disease such as systemic lupus erythematosus would be extremely low. We used self-reported arthritis as a measure to include anyone with RA symptoms and adjusted for individuals with self-reported arthritis as well as IL-6 levels in our analysis to account for potential confounding. Thus, we believe these results likely reflect persons without autoimmune disease. We recognize these exclusions are imperfect and that there may be residual confounding. Thus, these findings require external validation. While our focus was on the serum reactivity of individual antibodies to citrullinated proteins/peptides, anti-citrullinated antibody positivity is often defined clinically by the anti-CCP2 assay. When we evaluated the associations of anti-CCP2 with LV mass, LV ejection fraction, or risk of heart failure, however, results were similar. Considering the limitations of this study, it is the first to evaluate associations of serum reactivity to citrullinated protein/peptide antigens with myocardial structure and function in a community-living population without RA, and suggests that having extremely high reactivity to citrullinated protein/peptide antigens and subclinical CVD may be associated in the absence of clinical RA.

## Conclusion

In conclusion, approximately 27% of community-living individuals have serum reactivity to citrullinated protein/peptide antigens. These individuals appear similar in terms of demographic and clinical characteristics to those who have within normal reactivity to citrullinated antigens, with the exception of greater prevalence of anti-CCP2 antibodies and higher concentrations of the inflammatory biomarker IL-6. Overall, persons with extremely high reactivity to at least 1 citrullinated protein/peptide antigen had similar cardiac structure in terms of LVEF and LV mass, and similar risk for HF compared to those with normal reactivity to citrullinated protein/peptide antigens. Among the subset of 27% who had EHR antibodies, a greater number of EHR antibodies was independently associated with lower LVEF, however this did not appear to translate to higher risk of HF during approximately 11 years of follow-up. Further research is warranted to determine whether addressing autoimmune-related cardiovascular risk prior to clinical symptoms can prevent cardiac structural changes, particularly among those with greater number of EHR antibodies.

## Supporting information

S1 Table Complete list of antibodies to citrullinated and non-citrullinated protein/peptide antigens used in the array. (DOCX)

S2 Table Associations of antibodies to individual citrullinated and non-citrullinated protein/peptide antigens with LV ejection fraction and LV mass. (DOCX)

## Abbreviations

Anti-CCP2: Anti-cyclic Citrullinated Peptide
CVD: Cardiovascular Disease
EHR: Extremely High Reactivity; Extremely Highly Reactive
ELISA: Enzyme-linked Immunosorbent Assay
HF: Heart Failure
LV: Left Ventricle; Left Ventricular
LVEF: Left Ventricular Ejection Fraction
LVMI: Left Ventricular Mass Index
MESA: Multi-Ethnic Study of Atherosclerosis
RA: Rheumatoid Arthritis
RF: Rheumatoid Factor
WNR: Within Normal Reactivity

## References

[1] Nicola PJ, Maradit-Kremers H, Roger VL, Jacobsen SJ, Crowson CS, Ballman KV, et al. The risk of congestive heart failure in rheumatoid arthritis: a population-based study over 46 years. Arthritis Rheum. 2005;52(2):412–20. doi: 10.1002/art.20855 15692992

[2] Crowson CS, Nicola PJ, Kremers HM, O’Fallon WM, Therneau TM, Jacobsen SJ, et al. How much of the increased incidence of heart failure in rheumatoid arthritis is attributable to traditional cardiovascular risk factors and ischemic heart disease? Arthritis Rheum. 2005;52(10):3039–44. doi: 10.1002/art.21349 16200583

[3] Logstrup BB, Ellingsen T, Pedersen AB, Kjaersgaard A, Botker HE, Maeng M. Development of heart failure in patients with rheumatoid arthritis: A Danish population-based study. Eur J Clin Invest. 2018;48(5):e12915. doi: 10.1111/eci.12915 29464714

[4] Mantel A, Holmqvist M, Andersson DC, Lund LH, Askling J. Association Between Rheumatoid Arthritis and Risk of Ischemic and Nonischemic Heart Failure. J Am Coll Cardiol. 2017;69(10):1275–85. 28279294 10.1016/j.jacc.2016.12.033

[5] Myasoedova E, Crowson CS, Nicola PJ, Maradit-Kremers H, Davis JM 3rd, Roger VL, et al. The influence of rheumatoid arthritis disease characteristics on heart failure. J Rheumatol. 2011;38(8):1601–6. doi: 10.3899/jrheum.100979 21572155 PMC3337549

[6] Ahlers MJ, Lowery BD, Farber-Eger E, Wang TJ, Bradham W, Ormseth MJ, et al. Heart Failure Risk Associated With Rheumatoid Arthritis-Related Chronic Inflammation. J Am Heart Assoc. 2020;9(10):e014661. doi: 10.1161/JAHA.119.014661 32378457 PMC7660862

[7] Hughes-Austin JM, Young KA, Deane KD, Weisman MH, Buckner JH, Mikuls TR, et al., editors. Antibodies to citrullinated enolase, fibrinogen, and vimentin are associated with markers of endothelial dysfunction in first-degree relatives of patients with rheumatoid arthritis: the Studies of the Etiology of Rheumatoid Arthritis. ARTHRITIS AND RHEUMATISM; 2013: WILEY-BLACKWELL 111 RIVER ST, HOBOKEN 07030–5774, NJ USA.

[8] Hughes-Austin Jan M, Gan Ryan W, Deane Kevin D, Weisman Michael H, Demoruelle MK, Sokolove J, et al. Association of Antibodies to Citrullinated Protein Antigens with Blood Pressure in First-Degree Relatives of Rheumatoid Arthritis Patients: The Studies of the Etiology of Rheumatoid Arthritis. American Journal of Nephrology. 2017;46(6):481–7. doi: 10.1159/000485259 29237149 PMC5788181

[9] Smolen JS, Aletaha D, Barton A, Burmester GR, Emery P, Firestein GS, et al. Rheumatoid arthritis. Nat Rev Dis Primers. 2018;4:18001. doi: 10.1038/nrdp.2018.1 29417936

[10] Hughes-Austin JM, Deane KD, Derber LA, Kolfenbach JR, Zerbe GO, Sokolove J, et al. Multiple cytokines and chemokines are associated with rheumatoid arthritis-related autoimmunity in first-degree relatives without rheumatoid arthritis: Studies of the Aetiology of Rheumatoid Arthritis (SERA). Ann Rheum Dis. 2013;72(6):901–7. doi: 10.1136/annrheumdis-2012-201505 22915618 PMC3726193

[11] Libby P, Ridker PM, Maseri A. Inflammation and atherosclerosis. Circulation. 2002;105(9):1135–43. doi: 10.1161/hc0902.104353 11877368

[12] Golia E, Limongelli G, Natale F, Fimiani F, Maddaloni V, Pariggiano I, et al. Inflammation and cardiovascular disease: from pathogenesis to therapeutic target. Curr Atheroscler Rep. 2014;16(9):435. doi: 10.1007/s11883-014-0435-z 25037581

[13] Geraldino-Pardilla L, Russo C, Sokolove J, Robinson WH, Zartoshti A, Van Eyk J, et al. Association of anti-citrullinated protein or peptide antibodies with left ventricular structure and function in rheumatoid arthritis. Rheumatology (Oxford). 2017;56(4):534–40. doi: 10.1093/rheumatology/kew436 27994093 PMC5850788

[14] Demoruelle MK, Parish MC, Derber LA, Kolfenbach JR, Hughes-Austin JM, Weisman MH, et al. Performance of anti-cyclic citrullinated Peptide assays differs in subjects at increased risk of rheumatoid arthritis and subjects with established disease. Arthritis Rheum. 2013;65(9):2243–52. doi: 10.1002/art.38017 23686569 PMC3776020

[15] Majka DS, Deane KD, Parrish LA, Lazar AA, Baron AE, Walker CW, et al. Duration of preclinical rheumatoid arthritis-related autoantibody positivity increases in subjects with older age at time of disease diagnosis. Ann Rheum Dis. 2008;67(6):801–7. doi: 10.1136/ard.2007.076679 17974596 PMC3761074

[16] Rantapaa-Dahlqvist S, de Jong BA, Berglin E, Hallmans G, Wadell G, Stenlund H, et al. Antibodies against cyclic citrullinated peptide and IgA rheumatoid factor predict the development of rheumatoid arthritis. Arthritis Rheum. 2003;48(10):2741–9. doi: 10.1002/art.11223 14558078

[17] de Pablo P, Dietrich T, Chapple IL, Milward M, Chowdhury M, Charles PJ, et al. The autoantibody repertoire in periodontitis: a role in the induction of autoimmunity to citrullinated proteins in rheumatoid arthritis? Ann Rheum Dis. 2014;73(3):580–6. doi: 10.1136/annrheumdis-2012-202701 23434568

[18] Hueber W, Kidd BA, Tomooka BH, Lee BJ, Bruce B, Fries JF, et al. Antigen microarray profiling of autoantibodies in rheumatoid arthritis. Arthritis Rheum. 2005;52(9):2645–55. doi: 10.1002/art.21269 16142722

[19] Konig MF, Giles JT, Nigrovic PA, Andrade F. Antibodies to native and citrullinated RA33 (hnRNP A2/B1) challenge citrullination as the inciting principle underlying loss of tolerance in rheumatoid arthritis. Ann Rheum Dis. 2016;75(11):2022–8. doi: 10.1136/annrheumdis-2015-208529 26865600

[20] Quirke AM, Perry E, Cartwright A, Kelly C, De Soyza A, Eggleton P, et al. Bronchiectasis is a Model for Chronic Bacterial Infection Inducing Autoimmunity in Rheumatoid Arthritis. Arthritis Rheumatol. 2015;67(9):2335–42. doi: 10.1002/art.39226 26017630 PMC4832289

[21] Demoruelle MK, Bowers E, Lahey LJ, Sokolove J, Purmalek M, Seto NL, et al. Antibody Responses to Citrullinated and Noncitrullinated Antigens in the Sputum of Subjects With Rheumatoid Arthritis and Subjects at Risk for Development of Rheumatoid Arthritis. Arthritis Rheumatol. 2018;70(4):516–27. doi: 10.1002/art.40401 29266801 PMC5876113

[22] Tasliyurt T, Kisacik B, Kaya SU, Yildirim B, Pehlivan Y, Kutluturk F, et al. The frequency of antibodies against cyclic citrullinated peptides and rheumatoid factor in healthy population: a field study of rheumatoid arthritis from northern Turkey. Rheumatol Int. 2013;33(4):939–42. doi: 10.1007/s00296-012-2458-5 22829412

[23] Terao C, Ohmura K, Ikari K, Kawaguchi T, Takahashi M, Setoh K, et al. Effects of smoking and shared epitope on the production of anti-citrullinated peptide antibody in a Japanese adult population. Arthritis Care Res (Hoboken). 2014;66(12):1818–27. doi: 10.1002/acr.22385 24942650

[24] Bild DE, Bluemke DA, Burke GL, Detrano R, Diez Roux AV, Folsom AR, et al. Multi-ethnic study of atherosclerosis: objectives and design. Am J Epidemiol. 2002;156(9):871–81. doi: 10.1093/aje/kwf113 12397006

[25] Chandra PE, Sokolove J, Hipp BG, Lindstrom TM, Elder JT, Reveille JD, et al. Novel multiplex technology for diagnostic characterization of rheumatoid arthritis. Arthritis Res Ther. 2011;13(3):R102. doi: 10.1186/ar3383 21702928 PMC3218917

[26] Sokolove J, Bromberg R, Deane KD, Lahey LJ, Derber LA, Chandra PE, et al. Autoantibody epitope spreading in the pre-clinical phase predicts progression to rheumatoid arthritis. PLoS One. 2012;7(5):e35296. doi: 10.1371/journal.pone.0035296 22662108 PMC3360701

[27] Monach PA, Hueber W, Kessler B, Tomooka BH, BenBarak M, Simmons BP, et al. A broad screen for targets of immune complexes decorating arthritic joints highlights deposition of nucleosomes in rheumatoid arthritis. Proc Natl Acad Sci U S A. 2009;106(37):15867–72. doi: 10.1073/pnas.0908032106 19720992 PMC2747210

[28] Majka DS, Vu TT, Pope RM, Teodorescu M, Karlson EW, Liu K, et al. Association of Rheumatoid Factors With Subclinical and Clinical Atherosclerosis in African American Women: The Multiethnic Study of Atherosclerosis. Arthritis Care Res (Hoboken). 2017;69(2):166–74. doi: 10.1002/acr.22930 27159164

[29] Natori S, Lai S, Finn JP, Gomes AS, Hundley WG, Jerosch-Herold M, et al. Cardiovascular function in multi-ethnic study of atherosclerosis: normal values by age, sex, and ethnicity. AJR Am J Roentgenol. 2006;186(6 Suppl 2):S357–65. doi: 10.2214/AJR.04.1868 16714609

[30] Bluemke DA, Kronmal RA, Lima JA, Liu K, Olson J, Burke GL, et al. The relationship of left ventricular mass and geometry to incident cardiovascular events: the MESA (Multi-Ethnic Study of Atherosclerosis) study. J Am Coll Cardiol. 2008;52(25):2148–55. doi: 10.1016/j.jacc.2008.09.014 19095132 PMC2706368

[31] Fashanu OE, Upadhrasta S, Zhao D, Budoff MJ, Pandey A, Lima JAC, et al. Effect of Progression of Valvular Calcification on Left Ventricular Structure and Frequency of Incident Heart Failure (from the Multiethnic Study of Atherosclerosis). Am J Cardiol. 2020;134:99–107. doi: 10.1016/j.amjcard.2020.08.017 32917344 PMC7572881

[32] Mosteller RD. Simplified calculation of body-surface area. N Engl J Med. 1987;317(17):1098. doi: 10.1056/NEJM198710223171717 3657876

[33] Inker LA, Schmid CH, Tighiouart H, Eckfeldt JH, Feldman HI, Greene T, et al. Estimating glomerular filtration rate from serum creatinine and cystatin C. N Engl J Med. 2012;367(1):20–9. doi: 10.1056/NEJMoa1114248 22762315 PMC4398023

[34] Young KA, Deane KD, Derber LA, Hughes-Austin JM, Wagner CA, Sokolove J, et al. Relatives without rheumatoid arthritis show reactivity to anti-citrullinated protein/peptide antibodies that are associated with arthritis-related traits: studies of the etiology of rheumatoid arthritis. Arthritis Rheum. 2013;65(8):1995–2004. doi: 10.1002/art.38022 23754702 PMC3729718

[35] Arnett FC, Edworthy SM, Bloch DA, McShane DJ, Fries JF, Cooper NS, et al. The American Rheumatism Association 1987 revised criteria for the classification of rheumatoid arthritis. Arthritis Rheum. 1988;31(3):315–24. doi: 10.1002/art.1780310302 3358796

