# Supplementary material for: Serum reactivity to citrullinated protein/peptide antigens and left ventricular structure and function in the Multi-Ethnic Study of Atherosclerosis (MESA)
Source: PLoS One. 2023 Oct 24;18(10):e0291967. doi: 10.1371/journal.pone.0291967 (PMC10597499; doi:10.1371/journal.pone.0291967)
Supplement: S1 Table — (DOCX) [file pone.0291967.s001.docx]

**S1 SUPPLEMENTAL TABLE 1: Complete list of antibodies to citrullinated and non-citrullinated protein/peptide antigens used in the array**

| **Citrullinated Protein/Peptide Antigens** |
| --- |
| Apolipoprotein A1 _231-248_ |
| Apolipoprotein A1 |
| Apolipoprotein E _277-296 cyclic_ |
| Apolipoprotein E |
| Biglycan _247-266 cyclic_ |
| Clusterin _221-240 cyclic_ |
| Clusterin _231-250 cyclic_ |
| Enolase 1A _5-21_ |
| Fibrinogen B _54-74 cit 60,72,74_ |
| Fibrinogen |
| Fibrinogen A _211-230 cyclic_ |
| Fibrinogen A _27-43_ |
| Fibrinogen A _41-60 cyclic_ |
| Fibrinogen A _556-575 cyclic_ |
| Fibrinogen A _582-599_ |
| Fibrinogen A _616-635 cyclic_ |
| Fibrinogen B _246-267_ |
| Fibrinogen B _36-52_ |
| Fibronectin |
| Fibronectin _1035, 1036_ |
| Filaggrin _48-65 cyclic_ |
| Histone 2A |
| Histone 2A/a _1-20 cyclic_ |
| Histone 2A/a-2 _1-20_ |
| Histone 2B |
| Histone 2B/a _62-81 cyclic_ |
| Vimentin _1-16_ |
| Vimentin  **Non-Citrullinated Protein/Peptide Antigens**  Apolipoprotein A1  Apolipoprotein A1 _231-248_  Apolipoprotein E  Fibrinogen  Fibronectin  Filaggrin _48-65 arg2 v1 cyclic_  Histone 2A  Histone 2B  Tenascin C1  Tenascin C5 |
