# Supplementary material for: Serum reactivity to citrullinated protein/peptide antigens and left ventricular structure and function in the Multi-Ethnic Study of Atherosclerosis (MESA)
Source: PLoS One. 2023 Oct 24;18(10):e0291967. doi: 10.1371/journal.pone.0291967 (PMC10597499; doi:10.1371/journal.pone.0291967)
Supplement: S2 Table — (DOCX) [file pone.0291967.s002.docx]

**S2 SUPPLEMENTAL TABLE 2: Associations of antibodies to individual citrullinated and non-citrullinated protein/peptide antigens with LV ejection fraction and LV mass**

|  |  |  |  |  |
| --- | --- | --- | --- | --- |
| **Citrullinated Protein/Peptide Antigens** | **LV Ejection Fraction, %** | | **LV Mass, g** | |
|  | **B (SE)** | **p-value** | **B (SE)** | **p-value** |
| Apolipoprotein A1 _231-248_ | 0.0002 (0.0009) | 0.8451 | 0.002 (0.003) | 0.5797 |
| Apolipoprotein A1 | -0.00005 (0.004) | 0.9899 | 0.003 (0.02) | 0.8345 |
| Apolipoprotein E _277-296 cyclic_ | 0.0002 (0.0009) | 0.8457 | 0.002 (0.003) | 0.5014 |
| Apolipoprotein E | -0.003 (0.003) | 0.3065 | 0.01 (0.01) | 0.3058 |
| Biglycan _247-266 cyclic_ | -0.003 (0.001) | 0.746 | 0.002 (0.004) | 0.5001 |
| Clusterin _221-240 cyclic_ | -0.002 (0.001) | 0.0972 | 0.005 (0.004) | 0.2046 |
| Clusterin _231-250 cyclic_ | -0.0002 (0.001) | 0.8122 | 0.002 (0.004) | 0.556 |
| Enolase 1A _5-21_ | 0.00002 (0.001) | 0.9825 | 0.002 (0.004) | 0.5411 |
| Fibrinogen B _54-74 cit 60,72,74_ | -0.0002 (0.001) | 0.8445 | 0.002 (0.004) | 0.5549 |
| Fibrinogen | -0.001 (0.001) | 0.1668 | 0.005 (0.004) | 0.1855 |
| Fibrinogen A _211-230 cyclic_ | -0.0003 (0.0009) | 0.7528 | 0.002 (0.003) | 0.611 |
| Fibrinogen A _27-43_ | -0.0002 (0.0009) | 0.8339 | 0.002 (0.004) | 0.5726 |
| Fibrinogen A _41-60 cyclic_ | -0.0001 (0.001) | 0.8959 | 0.002 (0.004) | 0.5441 |
| Fibrinogen A _556-575 cyclic_ | -0.0008 (0.001) | 0.5856 | 0.002 (0.006) | 0.7138 |
| Fibrinogen A _582-599_ | -0.0001 (0.0009) | 0.8737 | 0.002 (0.003) | 0.5143 |
| Fibrinogen A _616-635 cyclic_ | -0.001 (0.0009) | 0.1516 | 0.004 (0.003) | 0.264 |
| Fibrinogen B _246-267_ | -0.002 (0.001) | 0.158 | 0.004 (0.004) | 0.3107 |
| Fibrinogen B _36-52_ | -0.002 (0.0009) | 0.1043 | 0.005 (0.003) | 0.1808 |
| Fibronectin | -0.003 (0.003) | 0.3337 | -0.002 (0.01) | 0.8932 |
| Fibronectin _1035, 1036_ | -0.002 (0.001) | 0.1289 | 0.004 (0.004) | 0.2821 |
| Filaggrin _48-65 cyclic_ | 0.0003 (0.0004) | 0.4337 | -0.0002 (0.002) | 0.9149 |
| Histone 2A | 0.0004 (0.0002) | 0.0679 | 0.0003 (0.0009) | 0.7662 |
| Histone 2A/a _1-20 cyclic_ | -0.001 (0.0007) | 0.0865 | 0.003 (0.003) | 0.2047 |
| Histone 2A/a-2 _1-20_ | -0.001 (0.001) | 0.1309 | 0.004 (0.004) | 0.2487 |
| **Histone 2B** | **-0.002 (0.0006)** | **0.005** | -0.001 (0.002) | 0.6872 |
| Histone _2B/a 62-81 cyclic_ | -0.002 (0.001) | 0.0761 | 0.006 (0.004) | 0.1548 |
| Vimentin _1-16_ | -0.002 (0.001) | 0.1299 | 0.005 (0.005) | 0.2306 |
| Vimentin _58-77 cyclic_ | -0.001 (0.001) | 0.1446 | 0.004 (0.004) | 0.2192 |
| Vimentin | -0.0013 (0.0009) | 0.1423 | 0.005 (0.003) | 0.177 |
| **Non-Citrullinated Protein/Peptide Antigens** | |  |  |  |
| Apolipoprotein A1 | 0.0002 (0.003) | 0.9535 | 0.002 (0.01) | 0.8958 |
| Apolipoprotein A1 _231-248_ | -0.001 (0.001) | 0.1505 | 0.005 (0.004) | 0.2057 |
| Apolipoprotein E | 0.008 (0.007) | 0.2626 | -0.011 (0.03) | 0.6758 |
| Fibrinogen | -0.001 (0.0009) | 0.1073 | 0.004 (0.003) | 0.2361 |
| Fibronectin | -0.002 (0.001) | 0.1379 | 0.004 (0.004) | 0.3372 |
| **Filaggrin _48-65 arg2 v1 cyclic_** | **-0.001 (0.0006)** | **0.0443** | 0.002 (0.002) | 0.4036 |
| **Histone 2A** | **-0.0008 (0.0004)** | **0.0383** | 0.0006 (0.001) | 0.7017 |
| **Histone 2B** | **-0.002 (0.0008)** | **0.0062** | -0.001 (0.003) | 0.6572 |
| Tenascin C1 | -0.00007 (0.001) | 0.956 | 0.001 (0.005) | 0.8506 |
| Tenascin C5 | -0.002 (0.001) | 0.1544 | 0.005 (0.004) | 0.2339 |
| Vimentin | -0.001 (0.0007) | 0.0978 | 0.004 (0.003) | 0.1754 |
|  |  |  |  |  |
| Adjusted for age, gender, race/ethnicity, smoking status, systolic blood pressure, use of anti-hypertensive medications, self-reported arthritis, IL-6, body surface area, and estimated glomerular filtration rate | | | | |
